# Ovarian Response in Urgent Fertility Preservation After Chemotherapy for Hematological Malignancies: Predictive Value of Anti-Müllerian Hormone and Antral Follicle Count

**DOI:** 10.3390/medicina62040666

**Published:** 2026-04-01

**Authors:** Yingqiao Ding, Yanling Wan, Tiantian Su, Jiajia Ai, Cheng Cheng, Yuan Fan, Li Tian

**Affiliations:** 1 Department of Obstetrics and Gynecology, Peking University People’s Hospital, Beijing 100044, China; dyq2011210274@163.com (Y.D.); wyl9208@163.com (Y.W.); sutiantian2019@163.com (T.S.);; 2 Reproductive Medical Center, Peking University People’s Hospital, Beijing 100044, China

**Keywords:** fertility preservation, chemotherapy, ovarian stimulation, hematological disease, oocyte cryopreservation, anti-Müllerian hormone

## Abstract

*Background and Objectives*: Fertility preservation in young patients with hematological malignancies is often constrained by the need for urgent life-saving chemotherapy, leaving limited evidence to guide counseling once treatment has already begun. Reliable predictors of ovarian response after chemotherapy are therefore clinically important. *Materials and Methods*: This retrospective single-center study included 37 hematological patients aged ≤35 years who underwent urgent controlled ovarian stimulation after initial chemotherapy. Only the first cycle per patient was analyzed. Patients were grouped by metaphase II oocyte yield as high-yield group (≥8 metaphase II oocytes) or low-yield group (<8). Post-chemotherapy ovarian reserve markers and chemotherapy-related variables were assessed. Parsimoniously adjusted logistic regression and ROC analyses were performed, and LOESS regression was used to explore relationships with mature oocyte number. *Results*: The median number of chemotherapy cycles before stimulation was three (IQR: 2–4), and the median interval from last chemotherapy to retrieval was 33 days (IQR: 27–39). The high-yield group had higher post-chemotherapy anti-Müllerian hormone (AMH) and antral follicle count (AFC) than the low-yield group (both *p* < 0.05). In adjusted analyses, AMH (OR 2.58, 95% CI 1.17–5.70) and AFC (OR 1.24, 95% CI 1.04–1.48) were associated with achieving ≥8 mature oocytes. No association was detected between oocyte yield and chemotherapy cycle number, chemotherapy-free interval, alkylating agent exposure, or stimulation-related factors. LOESS showed positive, non-linear associations for AMH and AFC with mature oocyte number. In this exploratory analysis, ROC curves suggested moderate discrimination for predicting high oocyte yield, with areas under the curve of 0.78 for AMH, 0.73 for AFC, and 0.80 for the combined model. *Conclusions*: Post-chemotherapy AMH and AFC were associated with ovarian response in urgent fertility preservation after initial chemotherapy for young hematological malignancies. Larger studies are needed to validate these exploratory findings.

## 1. Introduction

Hematological malignancies frequently affect females of reproductive age [1,2,3], and fertility preservation (FP) is often challenged by the need for immediate, non-deferrable chemotherapy, which is unlike patients with solid tumors. As a result, a substantial proportion of patients have already received chemotherapy by the time FP is considered. Approximately 46.0–48.7% of all types of hematologic patients have undergone at least one course of chemotherapy before FP [4,5]. According to data from our center, up to 89.9% of patients with hematological malignancies received chemotherapy before FP treatment. Fortunately, regimens commonly used for the initial chemotherapy of acute leukemia and lymphoma generally do not lead to immediate and permanent ovarian failure (POF) (<20%) [1,6,7]. However, chemotherapy-induced gonadotoxicity and the consequent decline in fertility have been established [8,9].

Women with hematological malignancies may not be eligible for standard FP options [6]. It is impractical for many patients to preserve fertility prior to gonadotoxic treatment due to the non-deferrable nature of initiating life-saving chemotherapy. Furthermore, a considerable proportion will require hematopoietic stem cell transplantation (HSCT) following initial induction and consolidation chemotherapy, and the attendant conditioning regimens are among the most gonadotoxic interventions, conferring an extremely high likelihood of POF and long-term infertility [10]. Therefore, the timely referral to a reproductive center for FP consultation is critical, as it may represent their only opportunity to preserve future fertility.

When FP is attempted after chemotherapy in hematological patients, clinical decision-making is particularly challenging. First, the time window available for FP intervention is extremely narrow, frequently restricted to a single menstrual cycle [5,11,12]. Controlled ovarian stimulation (COS) must be completed within this limited interval, which typically requires 10–12 days. Second, previous studies have reported high cancellation rates in post-chemotherapy stimulation cycles, raising concerns about the feasibility and efficiency of urgent intervention [13]. Consequently, inappropriate FP strategy may result in wasted time and potential delays in disease treatment. Evidence suggests that achieving at least eight metaphase II (MII) oocytes is associated with improved cumulative live birth outcomes in onco-fertility preservation populations [14]. Against this background, in hematologic patients who have already received chemotherapy, it becomes particularly important to recognize those who are still likely to achieve an adequate oocyte yield with urgent COS. Yet, data to support such decision-making are still scarce. Existing studies on ovarian stimulation outcomes have mainly focused on fertility preservation before chemotherapy initiation or included heterogeneous cancer populations. Data specifically addressing ovarian response after chemotherapy in hematological malignancies remain limited, particularly with respect to detailed chemotherapy-related parameters.

Therefore, this study focused on a real-world cohort of hematological patients undergoing urgent fertility preservation after chemotherapy, characterized by a short interval between chemotherapy exposure and oocyte retrieval. We aimed to identify post-chemotherapy predictors of mature oocyte yield in young patients with hematological malignancies to inform individualized counseling for those pursuing fertility preservation after chemotherapy.

## 2. Materials and Methods

### 2.1. Patients

This study retrospectively analyzed the clinical data of patients with hematological diseases who underwent COS for urgent FP shortly after chemotherapy from June 2016 to November 2025. The study was conducted in Peking University People’s Hospital (Beijing, China).

Inclusion criteria were as follows: (I) patients with hematological diseases (e.g., leukemia, lymphoma, myelodysplastic syndrome) confirmed by bone marrow aspiration and histopathological examination; (II) patients with strong desire for future fertility underwent urgent COS and transvaginal oocyte retrieval followed by cryopreservation of oocytes or embryos to preserve their fertility; (III) patients had received initial intravenous chemotherapy shortly before COS; (IV) myeloablative conditioning and HSCT had not been performed. Patients who underwent routine IVF for non-urgent FP purposes were excluded. Only the first stimulation cycle per patient was included in the analysis to avoid within-patient correlation. Only cycles in which oocyte retrieval was performed were included. Because cycle cancellation may be driven by non-biological factors, such as urgent HSCT scheduling, cancelled cycles were not combined with low-yield group in the primary analysis to avoid misclassifying biological ovarian response. And a total of 37 cycles from 37 patients were eventually included.

Based on the ovarian response to COS, patients were categorized into a low oocyte yield group (<8 MII oocytes, *n* = 20) and a high oocyte yield group (≥8 MII oocytes, *n* = 17). The threshold of 8 oocytes was selected based on previous research indicating that cryopreservation of ≥8 oocytes is associated with improved cumulative live birth outcomes of at least 35.8% in onco-fertility preservation patients under 35 years of age [14].

This study was approved by the Ethics Committee of Peking University People’s Hospital (reference number 2026PHB251-001) and performed in accordance with the Declaration of Helsinki. All patients had provided written informed consent for the use of their anonymized clinical data for research purposes at the time of treatment.

### 2.2. Variables

Clinical information, including age, body mass index (BMI), gestation and parity history, disease and chemotherapy history, was extracted from electronic medical records. Alkylating agent exposure was defined as receipt of any alkylating chemotherapeutic agent (e.g., cyclophosphamide) at any time prior to FP. Chemotherapy number was defined as the total number of intravenous chemotherapy sessions received including induction and consolidation chemotherapy. Chemotherapy-free days referred to the interval between the last day of intravenous chemotherapy and the day of oocyte retrieval. Retrieval day to HSCT denoted the interval from oocyte retrieval day to hematopoietic stem cell infusion day. Years after oocyte retrieval was defined as the time elapsed from the retrieval date to the present.

### 2.3. Fertility Preservation Treatment

Ovarian reserve was assessed after chemotherapy at the time of fertility counseling, including serum anti-Müllerian hormone (AMH), follicle-stimulating hormone (FSH), luteinizing hormone (LH), estradiol (E2) levels, and antral follicle count (AFC). AMH was measured in our hospital laboratory, which remained unchanged throughout the study period. AFC was defined as the total number of bilateral antral follicles measuring 2–10 mm in diameter. Transvaginal ultrasound was the preferred approach and transrectal ultrasound was used for unmarried patients or when transvaginal scanning was not feasible, which is performed by qualified reproductive ultrasound physicians.

COS followed routine clinical practice with dose adjustments based on ultrasound and hormonal monitoring. The stimulation protocol was determined according to the patient’s menstrual phase and clinician’s experience. Protocols were divided into gonadotropin-releasing hormone antagonist (GnRH-ant) protocol and others, including luteal phase ovarian stimulation (LPOS), progestin-primed ovarian stimulation (PPOS) and minimal stimulation protocol, as previously described [15,16]. Trigger medications consisted of human chorionic gonadotropin (hCG) alone (250 μg recombinant hCG (Merck Serono, Geneva, Switzerland) or 2000IU urinary hCG (LIVZON Pharmaceutical Group Inc., Zhuhai, China)), gonadotropin-releasing hormone agonist (GnRH-a (Ferring GmbH, Kiel, Germany)) alone (0.2 mg triptorelin), or a co-trigger medication combining hCG and GnRH-a. Oocyte retrieval was scheduled 34–36 h after trigger under ultrasound guidance. Retrieved MII oocytes were subsequently used for oocytes cryopreservation directly or in vitro fertilization/intracytoplasmic sperm injection (IVF/ICSI) followed by embryo cryopreservation for patients with a partner.

Ovarian stimulation parameters included the stimulation days, initial gonadotropin dose, and total gonadotropin dose. Serum levels of E2, progesterone (P), and LH were measured on the trigger day. The oocyte retrieval rate was calculated as the number of Oocyte–Corona–Cumulus Complexes (OCCCs) retrieved divided by the number of follicles aspirated. The maturation rate was defined as the number of MII oocytes divided by the number of OCCCs retrieved.

To improve interpretability, continuous variables with large units were rescaled and reported per 100 IU for gonadotropin dose and per 100 pg/mL for estradiol levels on trigger day.

### 2.4. Statistical Analysis

Continuous data with normal distribution are presented as mean ± SD and compared using Student’s t-test. Non-normally distributed continuous data are expressed as median (Q_1_, Q_3_) and compared using the Mann–Whitney U test. Categorical variables are presented as *n* (%) and compared using the chi-square test or Fisher’s exact test. Logistic regression analyses were performed to evaluate factors associated with mature oocyte yield and results are presented as odds ratios (ORs) and 95% confidence intervals (CIs). Given the limited sample size and number of events, a parsimonious adjustment strategy was prespecified to reduce the risk of overfitting. Receiver operating characteristic (ROC) curve analyses were conducted to assess the discriminative performance of factors. Exploratory cut-off values were identified by the Youden index. Scatter plots with locally estimated scatterplot smoothing (LOESS) were used to visualize the relationships between variables and the number of MII oocytes. LOESS curves with 95% confidence bands were applied for descriptive visualization of potential non-linear trends without assuming a specific functional form. Missing data were present for several variables and the exact number of observations included in the analyses is reported in Table 1, Table 2 and Table 3. Analyses were conducted using complete-case data for each comparison, and no imputation was performed. All analyses were performed using R software (version 4.3.0; http://www.r-project.org (accessed on 12 January 2026)). A two-sided *p* < 0.05 was considered statistically significant.

## 3. Results

### 3.1. Patients’ Clinical Characteristics

**Figure 1:**
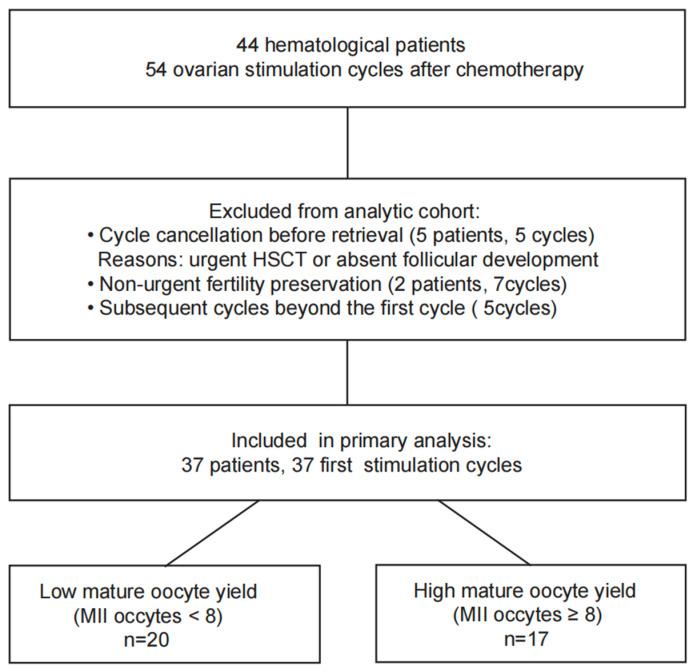
Study flowchart and analytic cohort.

A total of 44 patients with hematological diseases underwent 54 cycles of COS for urgent FP after chemotherapy. Seven cycles from two patients were excluded as they were performed for routine IVF treatment (≥1 year after chemotherapy when the disease was stable) rather than for urgent FP. Additionally, five cycles were canceled due to the urgent need for HSCT or absent follicular development, and detailed baseline characteristics are presented in Table S1. The majority of patients (32/37, 86.5%) underwent only one stimulation cycle, while four patients received two stimulation cycles and one patient received three stimulation cycles. Ultimately, data from the first stimulation cycle of 37 patients were included in the analysis (Figure 1).

All included patients were younger than 35 years old, with a mean age of 25.95 ± 3.83 years, ranging from 18 to 32. The median interval from disease diagnosis to FP was 0.33 years (IQR: 0.25, 0.50). The cohort predominantly consisted of patients with leukemia (89.19%), including both acute lymphoblastic leukemia (ALL) and acute myeloid leukemia (AML), followed by lymphoma (8.11%) and myelodysplastic syndrome (MDS) (2.70%). The median number of prior chemotherapies was three (IQR: 2, 4). The interval between the last day of chemotherapy and oocyte retrieval day ranged from 14 to 105 days, with a median of 33 days (IQR: 27.00, 39.00). Nineteen patients (54.29%) had alkylating agent exposure. The median interval from FP completion to hematopoietic stem cell infusion was 33 days (IQR: 26.00, 65.00) (Table 1).

**Table 1:** Demographic characteristics of hematologic patients after chemotherapy.

Variables	Total (*n* = 37)	Low Oocyte Yield (*n* = 20)	High Oocyte Yield (*n* = 17)	*p*
Age (years), Mean ± SD	25.95 ± 3.83	25.85 ± 4.06	26.06 ± 3.67	0.871
Range	18–32	18–32	21–32	/
BMI (kg/m^2^), Mean ± SD	22.24 ± 3.84	21.64 ± 3.43	22.95 ± 4.27	0.307
Disease Years, M (Q_1_, Q_3_)	0.33 (0.25, 0.50)	0.33 (0.25, 0.81)	0.25 (0.17, 0.50)	0.224
Diagnosis, *n* (%)				0.687
ALL	20 (54.05)	12 (60.00)	8 (47.06)	
AML	13 (35.14)	6 (30.00)	7 (41.18)	
Lymphoma	3 (8.11)	2 (10.00)	1 (5.88)	
MDS	1 (2.70)	0 (0.00)	1 (5.88)	
Gestation, *n* (%)				1.000
No	33 (89.19)	18 (90.00)	15 (88.24)	
Yes	4 (10.81)	2 (10.00)	2 (11.76)	
Parity, *n* (%)				0.489
No	35 (94.59)	18 (90.00)	17 (100.00)	
Yes	2 (5.41)	2 (10.00)	0 (0.00)	
Any alkylating agent exposure, *n* = 35, *n* (%)				0.095
No	16 (45.71)	6 (31.58)	10 (62.50)	
Yes	19 (54.29)	13 (68.42)	6 (37.50)	
Chemotherapy N, M (Q_1_, Q_3_)	3.00 (2.00, 4.00)	3.00 (2.00, 4.00)	3.00 (2.00, 4.00)	0.963
Chemotherapy-free days, M (Q_1_, Q_3_)	33.00 (27.00, 39.00)	32.50 (26.50, 38.25)	36.00 (29.00, 40.00)	0.417
Range	14–105	14–99	22–105	/
Retrieval day to HSCT (days), M (Q_1_, Q_3_)	33.00 (26.00, 65.00) (*n* = 29)	33.00 (26.25, 62.50) (*n* = 18)	33.00 (26.50, 75.50) (*n* = 11)	1.000

BMI, body mass index; ALL, acute lymphoblastic leukemia; AML, acute myeloid leukemia; MDS, myelodysplastic syndrome; HSCT, hematopoietic stem cell transplantation. The number of observations used for each variable is indicated in the table due to missing data.

Based on the number of MII oocytes retrieved, patients were categorized into a low oocyte yield group (MII < 8, *n* = 20) and a high oocyte yield group (MII ≥ 8, *n* = 17). Baseline characteristics were comparable between the two groups (Table 1). There were no significant differences in age, BMI, gestation and parity history, or disease diagnosis (*p* > 0.05). Furthermore, no significant differences were observed in the number of chemotherapy sessions or the chemotherapy-free days (*p* > 0.05). The distribution of alkylating agent exposure did not differ significantly between the two groups (*p* > 0.05).

Ovarian reserve assessments are shown in Table 2. The high oocyte yield group had significantly higher AMH (2.22 ± 1.43 vs. 0.98 ± 0.93, *p* = 0.009) and AFC (11.65 ± 4.53 vs. 7.65 ± 4.20, *p* = 0.009) levels compared to the low yield group.

**Table 2:** Post-chemotherapy ovarian reserve in hematologic patients.

Variables	Total (*n* = 37)	Low Oocyte Yield (*n* = 20)	High Oocyte Yield (*n* = 17)	*p*
AMH (ng/mL), Mean ± SD	1.62 ± 1.35 (*n* = 31)	0.98 ± 0.93 (*n* = 15)	2.22 ± 1.43 (*n* = 16)	**0.009**
FSH (IU/L), M (Q_1_, Q_3_)	7.10 (5.66, 11.53)	11.14 (6.29, 14.04)	6.21 (5.33, 7.25)	**0.039**
LH (IU/L), M (Q_1_, Q_3_)	5.22 (3.62, 8.85)	4.77 (3.30, 9.21)	5.45 (3.68, 8.85)	0.577
E2 (pg/mL), M (Q_1_, Q_3_)	52.60 (26.59, 127.17)	53.71 (26.47, 163.03)	49.98 (26.59, 106.82)	0.795
AFC, Mean ± SD	9.49 ± 4.74	7.65 ± 4.20	11.65 ± 4.53	**0.009**

AMH, Anti-Müllerian Hormone; FSH, follicle-stimulating hormone; LH, luteinizing hormone; E2, estradiol; AFC, antral follicle count. The number of observations used for each variable is indicated in the table due to missing data. Bold values indicate statistical significance (*p* < 0.05).

### 3.2. The Oocytes Retrieval Rate Was Low but the Maturation Rate Was Unaffected in the Low Oocyte Yield Group

Ovarian stimulation parameters are presented in Table 3. The initial gonadotropin dose was different between groups (*p* < 0.05). However, no significant differences were found in the duration of stimulation, total gonadotropin dose, stimulation protocol (*p* > 0.05). Trigger medication and hormone levels on the trigger day were also comparable (*p* > 0.05).

**Table 3:** Parameters of ovarian stimulation in hematologic patients after chemotherapy.

Variables	Total (*n* = 37)	Low Oocyte Yield (*n* = 20)	High Oocyte Yield (*n* = 17)	*p*
Years after oocyte retrieval, M (Q_1_, Q_3_)	2.00 (1.00, 6.00)	2.00 (1.00, 5.25)	3.00 (2.00, 6.00)	0.644
Days of stimulation, Mean ± SD	10.14 ± 2.38	9.55 ± 2.37	10.82 ± 2.27	0.106
Initial gonadotropin dose (per 100 IU), M (Q_1_, Q_3_)	2.25 (2.00, 3.00)	2.25 (2.25, 3.00)	2.12 (1.50, 2.25)	**0.036**
Total gonadotropin dose (per 100 IU), M (Q_1_, Q_3_)	24.00 (19.50, 30.00)	25.80 (23.06, 28.12)	20.75 (17.75, 33.00)	0.228
Stimulation protocol, *n* (%)				0.094
GnRH-ant	25 (67.57)	11 (55.00)	14 (82.35)	
Other	12 (32.43)	9 (45.00)	3 (17.65)	
E2 at trigger (per 100 pg/mL), M (Q_1_, Q_3_)	14.96 (5.09, 23.59) (*n* = 34)	10.66 (3.94, 18.01) (*n* = 17)	16.98 (8.71, 29.46) (*n* = 17)	0.079
P at trigger (ng/mL), M (Q_1_, Q_3_)	1.06 (0.70, 2.00) (*n* = 34)	1.20 (0.72, 2.11) (*n* = 17)	0.94 (0.70, 1.52) (*n* = 17)	0.480
LH at trigger (IU/L), M (Q_1_, Q_3_)	3.08 (1.70, 6.63) (*n* = 34)	5.05 (2.06, 11.15) (*n* = 17)	2.33 (1.58, 4.51) (*n* = 17)	0.092
Trigger medication				0.797
hCG only	14 (37.84)	7 (35.00)	7 (41.18)	
GnRH agonist only	4 (10.81)	3 (15.00)	1 (5.88)	
hCG + GnRH agonist	19 (51.35)	10 (50.00)	9 (52.94)	
Number of oocytes retrieved, Mean ± SD	8.95 ± 6.62	4.85 ± 3.07	13.76 ± 6.47	**<0.001**
Oocytes retrieval rate (%), Mean ± SD	66.84 ± 24.25	57.13 ± 26.88	78.27 ± 14.46	**0.005**
Number of mature oocytes, M (Q_1_, Q_3_)	6.00 (4.00, 9.00)	4.00 (1.75, 5.00)	9.00 (8.00, 11.00)	**<0.001**
Range	0–34	0–7	8–34	/
Maturation rate, M (Q_1_, Q_3_)	80.00 (62.50, 100.00)	66.67 (61.88, 100.00)	81.82 (73.68, 94.44)	0.447
FP strategy, *n* (%)				0.725
Oocyte cryopreservation	27 (72.97)	14 (70.00)	13 (76.47)	
Embryo cryopreservation	10 (27.03)	6 (30.00)	4 (23.53)	

GnRH-ant, gonadotropin-releasing hormone antagonist; E2, estradiol; P, progesterone; LH, luteinizing hormone; hCG, human chorionic gonadotropin; GnRH agonist, gonadotropin-releasing hormone agonist; FP, fertility preservation. The number of observations used for each variable is indicated in the table due to missing data. Bold values indicate statistical significance (*p* < 0.05).

Regarding oocyte retrieval outcomes, the high oocyte yield group had a significantly higher number of oocytes retrieved (13.76 ± 6.47 vs. 4.85 ± 3.07, *p* < 0.01) and a higher oocyte retrieval rate (78.27 ± 14.46% vs. 57.13 ± 26.88%, *p* = 0.005), consequently resulting in more mature oocytes (9.00 vs. 4.00, *p* < 0.01). However, the oocyte maturation rate was not significantly different between groups (*p* > 0.05).

### 3.3. Post-Chemotherapy AMH and AFC Are Associated with High Mature Oocyte Yield After Chemotherapy

Univariate logistic regression analysis identified post-chemotherapy AMH (OR: 2.58, 95% CI: 1.17, 5.70, *p* = 0.019) and AFC (OR: 1.24, 95% CI: 1.04, 1.48, *p* = 0.017) were associated with high oocyte yield (Table 4).

**Table 4:** Univariate analysis for high mature oocyte yield (≥8) in hematologic patients after chemotherapy.

Variables	High Mature Oocyte Yield (MII ≥ 8)	
	OR (95%CI)	*p*
Age (years)	1.01 (0.85, 1.20)	0.867
BMI (kg/m^2^)	1.10 (0.92, 1.31)	0.302
Diagnosis		
ALL	1.00 (Reference)	
AML	1.75 (0.43, 7.17)	0.437
Lymphoma	0.75 (0.06, 9.72)	0.826
MDS	/	/
Disease Years	0.73 (0.35, 1.54)	0.407
Any alkylating agent exposure		0.072
No	1.00 (Reference)	
Yes	0.28 (0.07, 1.12)	
Chemotherapy N	1.00 (0.75, 1.34)	0.978
Chemotherapy-free days	1.01 (0.98, 1.05)	0.514
AMH (ng/mL)	2.58 (1.17, 5.70)	**0.019**
FSH (IU/L)	0.85 (0.72, 0.99)	0.051
AFC	1.24 (1.04, 1.48)	**0.017**
Years after oocyte retrieval	1.03 (0.81, 1.31)	0.718
Days of stimulation	1.29 (0.94, 1.78)	0.116
Initial gonadotropin dose (per 100 IU)	0.27 (0.07, 1.06)	0.061
Total gonadotropin dose (per 100 IU)	1.00 (0.94, 1.06)	0.892
E2 at trigger (per 100 pg/mL)	1.05 (0.99, 1.12)	0.099
P at trigger (ng/mL)	0.85 (0.59, 1.25)	0.414
LH at trigger (IU/L),	0.80 (0.64, 1.01)	0.060
Stimulation protocol		
GnRH-ant	1.00 (Reference)	
Other	0.26 (0.06, 1.21)	0.085
Trigger medication		
hCG only	1.00 (Reference)	
GnRH agonist only	0.33 (0.03, 4.04)	0.388
hCG + GnRH agonist	0.90 (0.23, 3.58)	0.881

BMI, body mass index; ALL, acute lymphoblastic leukemia; AML, acute myeloid leukemia; MDS, myelodysplastic syndrome; AMH, Anti-Müllerian Hormone; FSH, follicle-stimulating hormone; AFC, antral follicle count; E2, estradiol; P, progesterone; LH, luteinizing hormone; GnRH-ant, gonadotropin-releasing hormone antagonist; hCG, human chorionic gonadotropin; GnRH agonist, gonadotropin-releasing hormone agonist. Bold values indicate statistical significance (*p* < 0.05).

Because AMH and AFC are correlated markers of ovarian reserve (Figure S1), they were evaluated in separate multivariable models to avoid collinearity. In addition, we used a parsimonious and prespecified strategy to minimize confounding while limiting overfitting and adjusted a priori for age and years after oocyte retrieval. After adjustment for prespecified covariates, AMH and AFC remained significantly associated with high oocyte yield (Table S2).

**Figure 2:**
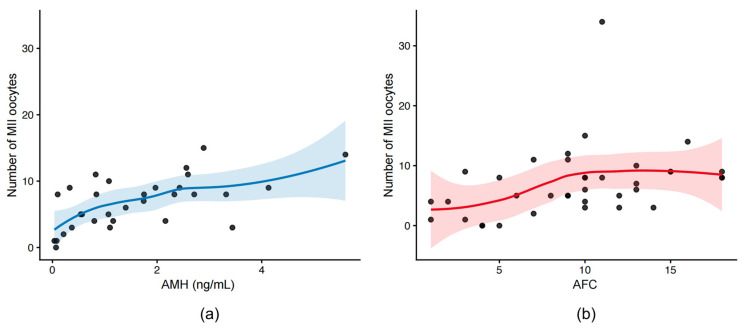
Relationships between post-chemotherapy ovarian reserve markers and the number of MII oocytes. (**a**) Serum AMH levels and MII oocyte number. (**b**) AFC and MII oocyte number. Scatter plots with LOESS smoothing and 95% confidence bands are shown.

LOESS regression demonstrated a positive, non-linear association between post-chemotherapy AMH levels and the number of MII oocytes. A similar pattern was observed for AFC, with higher AFC values corresponding to a higher mature oocyte yield. No clear threshold effect was evident across the observed ranges with AMH, suggesting a gradual increase in oocyte yield with increasing ovarian reserve markers (Figure 2a). The increase in MII number displayed a tendency to plateau at higher AFC values (Figure 2b).

**Figure 3:**
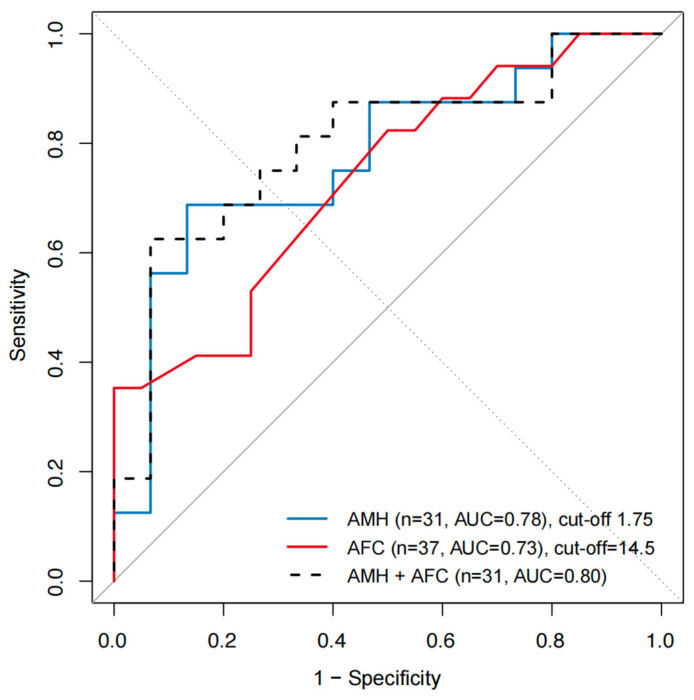
ROC curves for predicting high oocyte yield after chemotherapy. ROC curves showing the predictive performance of post-chemotherapy AMH, AFC, and their combination for achieving a high oocyte yield (≥8 MII oocytes). The AUC is presented for each model. Optimal cut-off values for AMH and AFC were identified using the Youden index. The solid grey diagonal line represents the line of no discrimination (AUC = 0.5). Analyses involving AMH were performed using cases with available AMH data (*n* = 31).

ROC analyses showed the discriminative ability of post-chemotherapy ovarian reserve markers for predicting high oocyte yield (≥8 MII oocytes). The area under the curve (AUC) was 0.78 for AMH (*n* = 31) and 0.73 for AFC (*n* = 37). A combined model showed a higher discrimination (AUC = 0.80, *n* = 31). Exploratory cut-off values identified using the Youden index were 1.75 ng/mL for AMH and 14.5 for AFC within this cohort. (Figure 3)

### 3.4. Chemotherapy-Related Variables Show No Detected Association with Mature Oocyte Yield

No significant associations were detected between mature oocyte yield and chemotherapy-related variables, including the number of chemotherapy sessions and the interval since the last chemotherapy (Table 4). LOESS analyses did not reveal a clear linear or threshold relationship between these variables and the number of mature oocytes, showing relatively flat trends across the observed ranges (Figure S2).

Alkylating agent exposure was comparable between two groups, and no association with oocyte yield was observed. Exposure to other chemotherapeutic classes, such as anthracyclines, vinca alkaloids, and antimetabolites, is summarized in Table S3.

In addition, ovarian stimulation parameters were not independently associated with mature oocyte yield in regression analyses. (Table 4)

## 4. Discussion

This study addresses an important and practical question in onco-fertility: counseling hematological patients who have already started chemotherapy and are considering urgent COS. Focusing on post-chemotherapy ovarian reserve assessment, we found that AMH and AFC were associated with achieving a high mature oocyte yield (≥8 MII oocytes). In contrast, no association was detected with the number of chemotherapy sessions, the chemotherapy-free interval, alkylating agent exposure, or stimulation protocol within this cohort.

Unlike many previous FP cohorts of hematological diseases, the present cohort was predominantly composed of patients with acute leukemia. This distribution reflects the clinical reality that acute leukemia patients often require urgent fertility preservation after initial chemotherapy prior to HSCT. Acute leukemia is also one of the main indications for HSCT. With advances in HSCT strategies and supportive care, the long-term prognosis is favorable among patients who remain relapse-free for two years after HSCT, with 10-year survival rates exceeding 80%. Therefore, the fertility needs of leukemia survivors are expected to increase, highlighting the importance of fertility preservation in this population.

The number of cryopreserved oocytes is closely associated with fertility outcomes in onco-fertility preservation. Cobo et al. reported that a yield of 8 oocytes corresponds to a cumulative live birth rate of 35.8%, whereas rates significantly dropped to only 9.1% when fewer than 5 oocytes are preserved [14,17]. While higher oocyte numbers (e.g., ≥15) have been proposed in social freezing populations, such thresholds may not be realistic in hematologic patients for urgent fertility preservation after chemotherapy. Based on this evidence, this study selected ≥8 MII oocytes as a clinically pragmatic endpoint. This study demonstrated post-chemotherapy AMH and AFC were significantly associated with high oocyte yield in hematological population after chemotherapy. And LOESS patterns were exploratory and supported a graded relationship between AMH/AFC and oocyte yield. This finding was consistent with previous studies in normal populations, which have established the predictive value of AMH for stimulation response and low oocyte yield [18,19,20,21,22]. This is also consistent the conclusion reported by Chan et al. that low AMH levels are associated with low oocyte production (<6) in onco-fertility population. Additionally, they found that a low AFC (≤6) was associated with cycle cancellation and reduced oocyte retrieval [13]. To our knowledge, this study is the first to definitively establish the association of AMH and AFC for ovarian response to stimulation in hematological population after chemotherapy. Additionally, although classification systems such as the Bologna or POSEIDON criteria have been proposed for infertile populations, their applicability in post-chemotherapy fertility preservation remains uncertain, as ovarian reserve markers may be transiently affected by recent treatment. And our findings extend existing evidence by demonstrating that ovarian reserve markers remain clinically informative even after recent chemotherapy exposure, a scenario that has been insufficiently studied.

The ROC findings suggest that post-chemotherapy AMH and AFC provide moderate discrimination for identifying patients more likely to achieve a high oocyte yield during urgent fertility preservation. However, given the limited sample size and lack of internal or external validation, the reported AUC estimates and data-derived cut-off values should be interpreted cautiously and regarded as hypothesis-generating rather than clinically definitive. Although this was a small cohort, it provides potentially useful data in a rare and challenging population and supports fertility preservation counseling before FP treatment initiation. In light of this finding, patients with lower post-chemotherapy AMH levels need to be informed about the possibility of poor COS outcomes and be provided with individualized FP options, such as ovarian tissue cryopreservation (OTC) and ovarian tissue oocyte in vitro maturation (OTO-IVM) [23,24,25]. These combined approaches aim to maximize the diversity of cryopreserved reproductive materials and improve long-term reproductive potential within the limited available time window.

The mechanisms by which chemotherapeutic agents impair ovarian are well-established, including the induction of DNA damage-triggered apoptosis in oocytes or granulosa cells, excessive activation and depletion of the primordial follicle pool, and disruption of ovarian microenvironment homeostasis [8,9]. It has been reported that when ovarian stimulation is performed before chemotherapy initiation, the oocyte yield in hematological patients is comparable to that of normal controls or patients with other malignancies [12,26,27,28]. Some studies even suggested lymphoma patients may perform better than controls [29]. Nevertheless, post-chemotherapy patients can still achieve a certain number of oocytes through COS [5,13]. Within the onco-fertility population, post-chemotherapy patients exhibit higher cycle cancellation rates and require higher doses of gonadotropins to achieve oocyte yields similar to those of pre-chemotherapy patients [13]. However, limited studies specifically within hematological populations have not conclusively demonstrated negative effects of prior chemotherapy on COS outcomes [4,5]. Existing literature on post-chemotherapy COS outcomes is characterized by significant heterogeneity in cancer types and control populations, and often lacks detailed chemotherapy-related parameters such as the interval since chemotherapy, the number of chemotherapy sessions and the classes of chemotherapeutic agents received. Therefore, we specifically focused on post-chemotherapy hematological patients to investigate whether such chemotherapy-related parameters influence the oocyte yield from urgent COS. Surprisingly, these chemotherapy-related factors showed no significant impact on the mature oocyte yield. Although our study population was relatively homogenous in terms of diagnosis, the diversity of chemotherapy regimens and individual variability in ovarian response to chemotherapy may have contributed to this negative result. Furthermore, a potential effect might still exist but remain undetected due to the small sample size and limited statistical power of this study.

To our knowledge, this is the first cohort of hematological malignancy patients reported to have the shortest time interval from last chemotherapy, with a median interval of only 33 days. Although the optimal timing for ovarian stimulation following chemotherapy remains unclear, FP interventions during the window between chemotherapy and HSCT have been described in hematological patients [30,31,32,33]. Initial chemotherapy regimens, distinct from myeloablative regimes, typically do not cause immediate POF, such as those commonly used for acute leukemia and lymphoma [1,6,7]. This provides a critical time window for referral and FP treatment after disease remission. However, data on reproductive outcomes using cryopreserved oocytes of hematological patients are scarce [27], and even less is known about the impact of recent chemotherapy exposure on gamete quality and offspring safety. Animal studies have indicated higher rates of miscarriage and birth defects in mice conceived shortly after chemotherapy exposure [34]. It was previously considered safe to cryopreserve oocytes or embryo for more than 6–12 months, but this is impractical for the urgent FP of hematologic patients. The American Society for Reproductive Medicine guidelines (2019, ASRM) explicitly state that the safe interval between completing chemotherapy and performing oocyte or embryo cryopreservation has not been established, emphasizing that human studies are needed to specifically examine the quality of oocytes and embryos resulting from prior chemotherapy [11]. Given that the ultimate goal of fertility preservation is live birth, we additionally followed three patients who returned to our reproductive center 4–6 years after HSCT to use oocytes obtained shortly after chemotherapy exposure. All three had acute leukemia and presented with markedly diminished ovarian reserve after HSCT. Two AML patients achieved pregnancy using oocytes retrieved approximately one month after chemotherapy, including one ongoing pregnancy and one preterm twin delivery without observed offspring abnormalities, whereas one ALL patient with prior alkylating agent exposure did not achieve pregnancy. The detailed information is listed in Table S4. Although limited by the small sample size, these observations suggest that oocytes obtained shortly after chemotherapy may retain developmental potential. However, long-term follow-up studies in this chemotherapy-exposure population are urgently needed to provide valuable insights. The preliminary findings of this study suggest that even when FP is initiated shortly after chemotherapy, regardless of the number of prior chemotherapy numbers, the chemotherapy-free intervals or diversity chemotherapy agents-exposure, patients with favorable AMH levels may still achieve an optimal oocyte yield through ovarian stimulation.

However, our study has several limitations. Given the special nature of the population and the rarity of the data, this was a retrospective study with a limited sample size, which restricts statistical power and the stability of multivariable estimates, and precluded internal validation of the predictive models. Missing data were handled using complete-case analyses. Therefore, bias due to non-random missingness cannot be excluded. Because cancelled cycles were not included in the primary cohort, our findings should be interpreted as associations with oocyte yield among cycles that reached oocyte retrieval, and selection bias remains possible. Additionally, detailed chemotherapy dosing data were unavailable for all patients, so we were unable to calculate cumulative exposure metrics such as cyclophosphamide equivalent dose. Instead, we focused on clinically accessible measures (post-chemotherapy AMH, chemotherapy sessions, and chemotherapy-free interval) that are routinely available at fertility counseling. Future studies with more complete regimen and dose information are warranted. Finally, most oocytes or embryos remain cryopreserved, and pregnancy outcomes as well as long-term offspring safety following the use of post-chemotherapy oocytes could not be evaluated in this study. Despite these limitations, this study provides clinically relevant evidence in a real-world post-chemotherapy setting that has been insufficiently addressed in previous literature. Larger multicenter prospective studies with longer follow-up are needed to assess reproductive and offspring outcomes in hematological patients undergoing FP after chemotherapy.

## 5. Conclusions

This study found that post-chemotherapy AMH and AFC were associated with achieving a high mature oocyte yield (≥8 MII oocytes) during urgent FP in young patients with hematological malignancies, whereas no association between chemotherapy-related variables and oocyte yield was detected within this cohort. For patients with low AMH or AFC, counseling should address the likelihood of a suboptimal ovarian response and lower oocyte yield, and consider individualized alternative FP strategies.

## Data Availability

The data presented in this study are available on reasonable request from the corresponding author.

## Supplementary Materials

The following supporting information can be downloaded at: https://www.mdpi.com/article/10.3390/medicina62040666/s1, Figure S1: Exploratory relationships between chemotherapy-related variables and MII oocyte number; Figure S2: Correlation between AMH and AFC.; Table S1: Baseline characteristics and reasons for cycle cancellation before retrieval; Table S2: Logistic regression models evaluating the relationship between factors and high mature oocyte yield (≥8) in hematologic patients after chemotherapy; Table S3: Chemotherapy exposure prior to ovarian stimulation; Table S4: Clinical characteristics and reproductive outcomes of patients who returned to use cryopreserved materials.

## Abbreviations

The following abbreviations are used in this manuscript:

FP	Fertility preservation
POF	Permanent ovarian failure
HSCT	Hematopoietic stem cell transplantation
COS	Controlled ovarian stimulation
MII	metaphase II
BMI	Body mass index
AMH	Anti-Müllerian hormone
FSH	Follicle-stimulating hormone
LH	Luteinizing hormone
E2	Estradiol
AFC	Antral follicle count
GnRH-ant	Gonadotropin-releasing hormone antagonist
LPOS	Luteal phase ovarian stimulation
PPOS	Progestin-primed ovarian stimulation
hCG	Human chorionic gonadotropin
P	Progesterone
OCCCs	Oocyte-Corona-Cumulus Complexes
OR	Odds ratio
CI	Confidence interval
ROC	Receiver operating characteristic
LOESS	Locally estimated scatterplot smoothing
ALL	Acute lymphoblastic leukemia
AML	Acute myeloid leukemia
MDS	Myelodysplastic syndrome
AUC	Area under the curve
OTC	Ovarian tissue cryopreservation
OTO-IVM	Ovarian tissue oocyte in vitro maturation
