# Supplementary material for: Ovarian Response in Urgent Fertility Preservation After Chemotherapy for Hematological Malignancies: Predictive Value of Anti-Müllerian Hormone and Antral Follicle Count
Source: Medicina (Kaunas). 2026 Apr 1;62(4):666. doi: 10.3390/medicina62040666 (PMC13118262; doi:10.3390/medicina62040666)
Supplement: Supplementary file 1 [file medicina-62-00666-s001.zip › Figure S1.pdf]

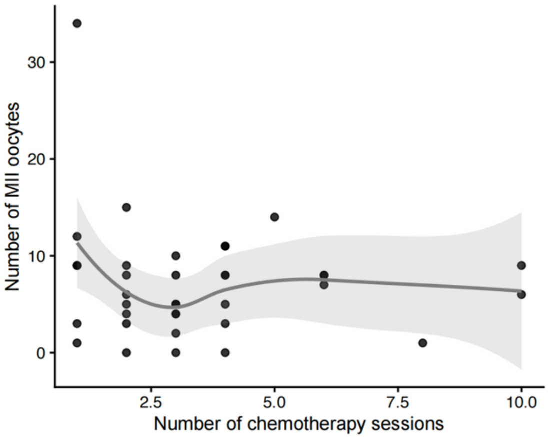

(a)

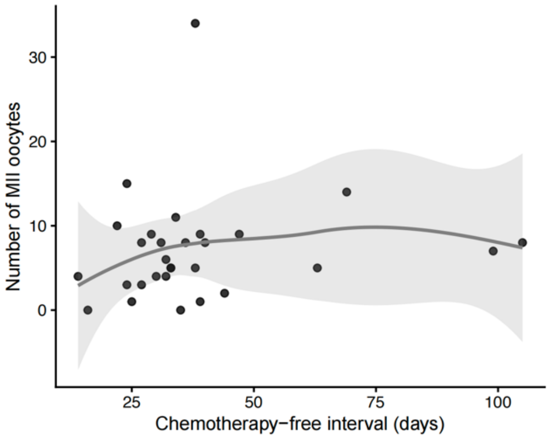

(b)

Figure S1. Exploratory relationships between chemotherapy-related variables and MII oocyte number. (a) Number of chemotherapy sessions and MII oocyte number. (b) Chemotherapy-free interval days and MII oocyte number. Scatter plots with LOESS smoothing and 95% confidence bands are shown.
