# Supplementary material for: Ovarian Response in Urgent Fertility Preservation After Chemotherapy for Hematological Malignancies: Predictive Value of Anti-Müllerian Hormone and Antral Follicle Count
Source: Medicina (Kaunas). 2026 Apr 1;62(4):666. doi: 10.3390/medicina62040666 (PMC13118262; doi:10.3390/medicina62040666)
Supplement: Supplementary file 1 [file medicina-62-00666-s001.zip › TableS1.pdf]

Table S1. Baseline characteristics and reasons for cycle cancellation before retrieval.

Variables	Cancelled cycles
**Patients, n**	5
**Cycles, n**	5
Age (years), M (Q_1, Q_3)	25 (23, 25)
Diagnosis, N (%)	
ALL	4 (80%)
AML	1 (20%)
AMH (ng/ml), M (Q_1, Q_3)	0.63 (0.10, 2.59)
AFC, M (Q_1, Q_3)	5 (5, 7)
**Reason for cycle cancellation, n (%)**	
Urgent HSCT	2 (40%)
Absent or insufficient follicular development	3 (60%)

ALL, acute lymphoblastic leukemia; AML, acute myeloid leukemia; AMH, Anti-Müllerian Hormone; AFC, antral follicle count; HSCT: hematopoietic stem cell transplantation.
