# Supplementary material for: Ovarian Response in Urgent Fertility Preservation After Chemotherapy for Hematological Malignancies: Predictive Value of Anti-Müllerian Hormone and Antral Follicle Count
Source: Medicina (Kaunas). 2026 Apr 1;62(4):666. doi: 10.3390/medicina62040666 (PMC13118262; doi:10.3390/medicina62040666)
Supplement: Supplementary file 1 [file medicina-62-00666-s001.zip › TableS2.pdf]

Table S2. Logistic regression models evaluating the relationship between factors and high mature oocyte yield (≥8) in hematologic patients after chemotherapy

**Variables**	**unadjusted (OR, 95%CI)** ***P***	**Model I (OR, 95%CI)** ***P***	**Model II (OR, 95%CI)** ***P***
AMH (ng/ml)	2.58 (1.17 ~ 5.70) **0.019**	2.67 (1.18 ~ 6.05) **0.019**	3.36 (1.25 ~ 9.02) **0.016**
AFC	1.24 (1.04 ~ 1.48) **0.017**	1.26 (1.05 ~ 1.52) **0.015**	1.26 (1.05 ~ 1.53) **0.016**

AMH, Anti-Müllerian Hormone; AFC, antral follicle count. Non-adjusted model adjusted model for none; Model I Adjusted for: Age; model II Adjusted for: Age, Years after oocyte retrieval.
