# Supplementary material for: Ovarian Response in Urgent Fertility Preservation After Chemotherapy for Hematological Malignancies: Predictive Value of Anti-Müllerian Hormone and Antral Follicle Count
Source: Medicina (Kaunas). 2026 Apr 1;62(4):666. doi: 10.3390/medicina62040666 (PMC13118262; doi:10.3390/medicina62040666)
Supplement: Supplementary file 1 [file medicina-62-00666-s001.zip › TableS3.pdf]

Table S3. Chemotherapy exposure prior to ovarian stimulation.

**Variables**	**Patients, *n* (%) (*n* = 35)**
**Chemotherapeutic agents ever exposure (non–mutually exclusive)**	
Any alkylating agent	
Cyclophosphamide	19 (54.29)
Ifosfamide	0 (0)
Busulfan	0 (0)
Procarbazine	0 (0)
Anthracyclines	27 (77.14)
Antimetabolites	23 (65.71)
Vinca alkaloids	20 (57.14)
Other agents	11 (31.43)
**Overall chemotherapy exposure category (mutually exclusive)**	
Alkylator-containing regimens (non-conditioning)	19 (54.29)
Non-alkylator regimens	16 (45.71)

Chemotherapy exposure was summarized at the patient level. Ever exposure was defined as receipt of a given agent or class at any time prior to ovarian stimulation. Patients may have received more than one chemotherapeutic agent; therefore, percentages in the non–mutually exclusive section do not sum to 100%. Percentages were calculated using the number of patients with available chemotherapy regimen details as the denominator (n=35). Other agents included chemotherapeutic drugs not categorized under the predefined major classes.
