# Supplementary material for: Ovarian Response in Urgent Fertility Preservation After Chemotherapy for Hematological Malignancies: Predictive Value of Anti-Müllerian Hormone and Antral Follicle Count
Source: Medicina (Kaunas). 2026 Apr 1;62(4):666. doi: 10.3390/medicina62040666 (PMC13118262; doi:10.3390/medicina62040666)
Supplement: Supplementary file 1 [file medicina-62-00666-s001.zip › TableS4.pdf]

Table S4. Clinical characteristics and reproductive outcomes of patients who returned to use cryopreserved materials.

Pt	Age (years)	Diagnosis	Chemo-therapy N	Chemo-therapy free days	Alkylating agent exposure	Fertilization Method	Embryo Transfer	Interval from HSCT to Transfer (years)	AMH after HSCT (ng/mL)	Outcome
1	29	ALL	3	33	Yes	IVF	2 embryos	4	0.01	Failed
2	28	AML	10	30	No	ICSI	2 embryos	6	0.02	Preterm twins
3	24	AML	4	35	No	ICSI	2 embryos	6	0.01	Ongoing pregnancy

ALL, acute lymphoblastic leukemia; AML, acute myeloid leukemia; IVF, in vitro fertilization; ICSI, intracytoplasmic sperm injection.
